# Oxidative Stress, Cytotoxicity and Genotoxicity in Earthworm *Eisenia fetida* at Different Di-*n*-Butyl Phthalate Exposure Levels

**DOI:** 10.1371/journal.pone.0151128

**Published:** 2016-03-16

**Authors:** Tingting Ma, Li’ke Chen, Longhua Wu, Haibo Zhang, Yongming Luo

**Affiliations:** 1 Institute of Hanjiang, Hubei University of Arts and Science, Xiangyang, China; 2 Key Laboratory of Soil Environment and Pollution Remediation, Institute of Soil Science, Chinese Academy of Sciences, Nanjing, China; 3 Shanghai Research Institute of Chemical Industry, Shanghai, China; 4 Key Laboratory of Coastal Zone Environmental Processes, Yantai Institute of Costal Zone Research, Chinese Academy of Sciences, Yantai, China; Jinling Institute of Technology, CHINA

## Abstract

Recognized as ubiquitous contaminants in soil, the environmental risk of phthalic acid esters (PAEs) is of great concern recently. Effects of di-*n*-butyl phthalate (DnBP), an extensively used PAE compound to *Eisenia fetida* have been investigated in spiked natural brown yellow soil (Alfisol) for soil contact test. The toxicity of DnBP to *E*. *fetida* on the activity of superoxide dismutase (SOD) activity, peroxidase (POD), reactive oxygen species (ROS) content, and the apoptosis of coelomocytes and DNA damage at the 7th, 14th, 21st and 28th day of the incubation have been paid close attention to. In general, SOD activity and ROS content were significantly induced, opposite to total protein content and POD activity, during the toxicity test of 28 days especially under concentrations higher than 2.5 mg kg^-1^. The reduction in neutral red retention (NRR) time along with the increase of dead coelomocytes as the increasing of DnBP concentrations, indicating severe damage to cell viability under varying pollutant stress during cultivation, which could also be proved by comet assay results for exerting evident DNA damage in coelomocytes. DnBP in spiked natural soil could indeed cause damage to tissues, coelomocytes and the nucleus of *E*. *fetida*. The key point of the apparent change in different indices presented around 2.5 mg DnBP kg^-1^ soil, which could be recommended as the threshold of DnBP soil contamination, so that further investigation on threshold values to other soil animals or microorganisms could be discussed.

## Introduction

China is one of the largest consumers of phthalic acid esters (PAEs), mainly used as plasticizers, and the consumption of PAEs has been much more than over 0.87 million tons per year after 2006 [1]. Emerging as synthetic environmental organic pollutants with high molecular mass, PAE compounds have been introduced to different environmental matrices worldwide, resulting in damage of non-target organisms in various ecosystems including agricultural plants, soil animals and microorganisms in the soil ecosystem [2, 3]. After a half century of supposedly safe use, the impacts of PAEs on ecological systems and human health raised serious concerns because of the adverse effects on animal cells [4]. In 1999, six representative PAE compounds, including di-*n*-butyl phthalate (DnBP), were nominated as priority pollutants and environmental endocrine disruptor chemicals (EDCs) by the Environmental Protection Agency of the United States (US EPA). The rising trend of DnBP in soils throughout China at concentrations from 0.24 mg kg^-1^ to about 10 mg kg^-1^ is worrisome these days [5] so that it has been listed as priority control pollutants in China. High residual concentrations in soil and slow degradation rate of DnBP have made the upcoming phytotoxicity problem to crops receive more public concern in recent years [6, 7], however, the possibility of being transferred from soils to human body and cause potential reproductive and developmental toxicity highlighted the importance of evaluating DnBP contamination in soils [8].

Earthworms comprise the largest part of the soil fauna biomass and they have been employed and proved as one of the most sensitive bio-indicators in the evaluation of chemical toxicity in soil. They play an important role in many soil-forming processes and also serve as a substantial food source for several higher organisms, such as birds and moles [9]. Although both amoebocytes and eleocytes are coelomocytes populations, only amoebocytes are the major attention of this test for all mitotic figures observed in them [10]. To investigate the potential toxicity effects of DnBP to *Eisenia fetida*, the natural spiked soil contact method, instead of the artificial soil method, has been adopted for the reliability in practical environment for the microscopic alteration on tissue and cellular levels [11]. Changes in anti-oxidant activity in toxicity tests at medium pollutant concentrations must occur to repair the induced trauma and to prevent further damage, so that they have been utilized as reliable regular indices for toxicity test on individual level [12]. On a cellular level, flow cytometry technique has exhibited substantial advantages in revealing the detailed alternations in the coelomocytes of earthworms since last century [13]. As a result, in testing cytotoxicity to earthworm coelomocytes, the viability of cell membranes as well as synthesis of intracellular DNA and proteins can be monitored [14]. The comet assay often led to more sensitive, reliable and novel conclusions in investigating genotoxicity effects of polluted soils to earthworms [13, 15, 16]. Among all the resorted parameters for quantification of DNA damage, length of tail shows the migration of cell nucleus caused by hazardous substances, which indicates both the DNA damage level and the length of broken DNA fragments; tail DNA % may accurately reflect the true levels of DNA damage because under different concentrations of the same target pollutants, the content of DNA in the tail had positive relation with DNA damage. Tail moment (TM) merges tail DNA density value multiplied by the migration distance and olive tail moment (OTM) indicates electrophoretic mobility of DNA induced by DNA damage [17, 18]. The neutral red retention (NRR) assay is one of the most popular earthworm biomarkers. It combines analytical simplicity and ecological realism (complexity) to the measurement of lysosomal membrane stability, and it supplementary describes the mechanisms of the pollutant in doing damage to target earthworms in the spiked soil test [19].

In the present study, for the evaluation of tissue, cellular and gene toxicity of DnBP to *E*. *fetida* at different exposure concentrations during 28 days in practical soils, the activities/contents of total protein, SOD, POD and ROS levels, coelomocyte cell apoptosis and NRR time analysis, together with the nuclear DNA damage and tailing were determined and compared in the spiked soil. The threshold concentration of DnBP contamination in natural soil and sensitive biomarkers were also discussed.

## Materials and Methods

### Ethics statement

This experiment did not involve any endangered or protected species. The earthworms were purchased from Dachang District of Nanjing, Jiangsu Province, China.

### Chemicals and Reagents

DnBP (99.1%) was obtained from AccuStandard^®^ Incorporation (New Haven, CT). All the other chemicals (reagent grade) were purchased from Sigma Chemical Corporation (St. Louis, MO) and the National Pharmaceutical Group Chemical Reagent Limited Corporation (Shanghai, China). Assay kits (Catalog A045-3 for BCA/ total protein content, A001-1 for SOD, A084-1 for POD and E004 for ROS) were from Nanjing Jiancheng Bioengineering Institute (Nanjing, China). Annexin V conjugated with fluorescein and propidium iodide (AV-FITC/PI) apoptosis detection kits were sourced from BD Biosciences Pharmingen (San Diego, CA).

### Toxicological Test

The toxicity tests on earthworms (*E*. *fetida*) were conducted in natural soil gathered from the top 15 cm of a seldom disturbed mountain area (118°57'57.83"E, 32°8'50.90"N) at Qixia District, in Nanjing, Jiangsu province, east China. It is a typical yellow brown soil classified as Alfisols according to the USDA soil classification with a pH (in water) of 7.4, a clay content of 1.67 g kg^-1^, an organic matter content of 14.6 g kg^-1^, and available nitrogen, phosphorus and potassium concentrations of 96.8, 14.4 and 102.8 mg kg^-1^, respectively. The soil was passed through a 2 mm sieve prior to the incubation test and the background concentrations of DnBP was determined to be 36.6 ± 2.4 μg kg^-1^.

Every 1000-g aliquot of soil was weighed and placed in a brown reagent jar after a stock solutions of DnBP dissolved in acetone were sprayed on the surface of the test soil and thoroughly mixed until the evaporation of 5 mL acetone solution to give final concentrations of about 1, 2.5, 5 and 10 mg DnBP kg^-1^ soil in the different treatments. The control treatment (0 mg kg^-1^) was sprayed with 5 mL of pure acetone and mixed the same as other treatments to ensure that toxicity was not due to the solvent. Four replicate jars were employed under each DnBP concentration and 20 worms were placed into each replicate jar, implying the total number of test worms were 400, before incubation under the same conditions. During the incubation, four earthworms were collected from each replicate jar for enzyme analysis, flow cytometry determination and the comet assay on the 7th, 14th, 21st, and 28th day respectively.

### DnBP quantitative analysis

The analysis of DnBP concentrations in soil after the incubation of 28 days were carried out following the approaches of Ma et al. [20].

### Harvest of samples and determination

The earthworms were placed in a pre-chilled glass homogenizer and ground in ice-cold phosphate buffered saline (PBS) (1:9, w/v, pH 7.5, containing 0.2 g L^-1^ KCl, 0.9% NaCl, 0.24 g L^-1^ KH_2_PO_4_, 1.44 g L^-1^ Na_2_HPO_4_ and 1 mM EDTA). The homogenate was centrifuged at 8000 rpm for 20 min at 4°C and the supernatant was kept at 4°C before used for the analysis of activity/content of the total protein, and several enzymes.

The coelomocytes were harvested using the non-invasive extrusion method described by Eyambe et al. [21]. Individual earthworms were first placed in 1.5-mL Eppendorf tubes and rinsed with 1 mL of PBS for 3 min twice to remove the mucus and feculence of the worm surface. Then 1 mL of extrusion medium which composed of 95% normal saline, 5% ethanol, 10 mg mL^-1^ guaiacol glyceryl ether and 2.5 mg mL^-1^ EDTA (pH 7.3), was added to stimulate spontaneous release of celomocytes. After disposal of the remaining worm bodies, the extrusion medium was centrifuged at 8000 rpm at 4°C and the precipitate was washed with the same PBS (pH 7.5) and centrifuged three times to collect the amoebocytes prior to flow cytometry analysis and comet assay. All procedures for enzyme solution and coelomocytes preparation were carried out at about 4°C to protect both enzyme activity and cells.

#### Antioxidant determination

The total protein content, activity of SOD, POD and ROS content were performed according to the instructions in kits (A045-3, A001-1, A084-1 and E004).

#### Neutral-red retention time (NRRT) assay

The measurement and counting of the neutral-red retention time, recording the time that half number of the cells were stained red, was following the method described by Weeks and Svendsen [22].

#### Double-labeled analysis of cell apoptosis rate

Apoptotic cell death was analyzed by determination of the loss of membrane phospholipid symmetry and membrane integrity [23] using an AV-FITC/PI apoptosis detection kit (Becton Dickinson Biosciences, United States) following the manufacturer's protocol. Cells were re-suspended at a final concentration of about 2 × 10^6^ cells mL^-1^ for 500 μL and transferred into a siliconized polypropylene tube before they were centrifuged and washed with Annexin V binding buffer (51-66121E, Becton, Dickinson and Company/BD, San Jose, CA, USA). The binding of cells was performed after incubating for 20 min at 4°C. Then 10 μL of both AV-FITC and PI were added and incubated for 15 min at room temperature to distinguish between live (AV-/PI-), apoptotic (AV+/PI-) and necrotic cells (AV+/PI+) or cell debris (AV-/PI+). After staining, the AV-FITC/PI double-stained cells were analyzed using a FACScan Flow Cytometer (BD, USA) equipped with CellQuest software (BD, USA). The parameters of the measurement were set at SSC 480; FL1 510; FL2 500, and a total 10 000 cells were counted. Eleocytes could be removed to a really low level in the procedure of amoebocytes harvest by washing and centrifuging and the removal effect could be read in the monitoring of CK in the forth quadrant for their autofluorescence intensity.

#### The comet assay

The comet assay was performed according to Song et al. [24]. The fluorescence microscopy analysis were performed with Zeiss Axiovert 40 (Carl Zeiss AG, Oberkochen, Germany) using a Charge Coupled Device (CCD) digital imaging system. The images of the comet assay were analyzed last using CASP [25]. Every 100 cell nuclei on each slide were analyzed. Length of tail, tail DNA ratio, tail moment and Olive tail moment were used for the quantification of DNA damage. From repeated experiments the average median tail moment value was calculated for each treatment group from the median tail moment value of each slide.

### Statistical Analysis

All values are presented as mean ± standard error of the mean (SEM). One-way analysis of variance and the least significant difference test were performed using the SPSS software (SPSS 13.0) package and *p< 0.05 and **p<0.01 were considered to be significant and highly significant probability levels, respectively.

## Results and Discussion

### Residual of DnBP in soils

According to the reported concentrations of DnBP in farmland soils in China, which are generally below 10 mg DnBP kg^-1^ soil (dry weight/ DW), the maximum concentration of this experiment was selected. After the incubation of 28 days, the residual concentrations of DnBP in different treatment have been detected to be 36.6 ± 2.4, 203.5 ± 3.8, 1139.3 ± 5.9, 2257.2 ± 4.3 and 4487.6 ± 9.2 μg kg^-1^ in treatment of 0, 1000, 2500, 5000 and 10000 μg DnBP kg^-1^ soil. DnBP in all treatments have been degraded to less than half of the initial concentrations, which indicated that the toxicity effect of DnBP continue all along.

### Biochemistry parameters analysis in spiked soil

#### Total protein content

**Fig 1:**
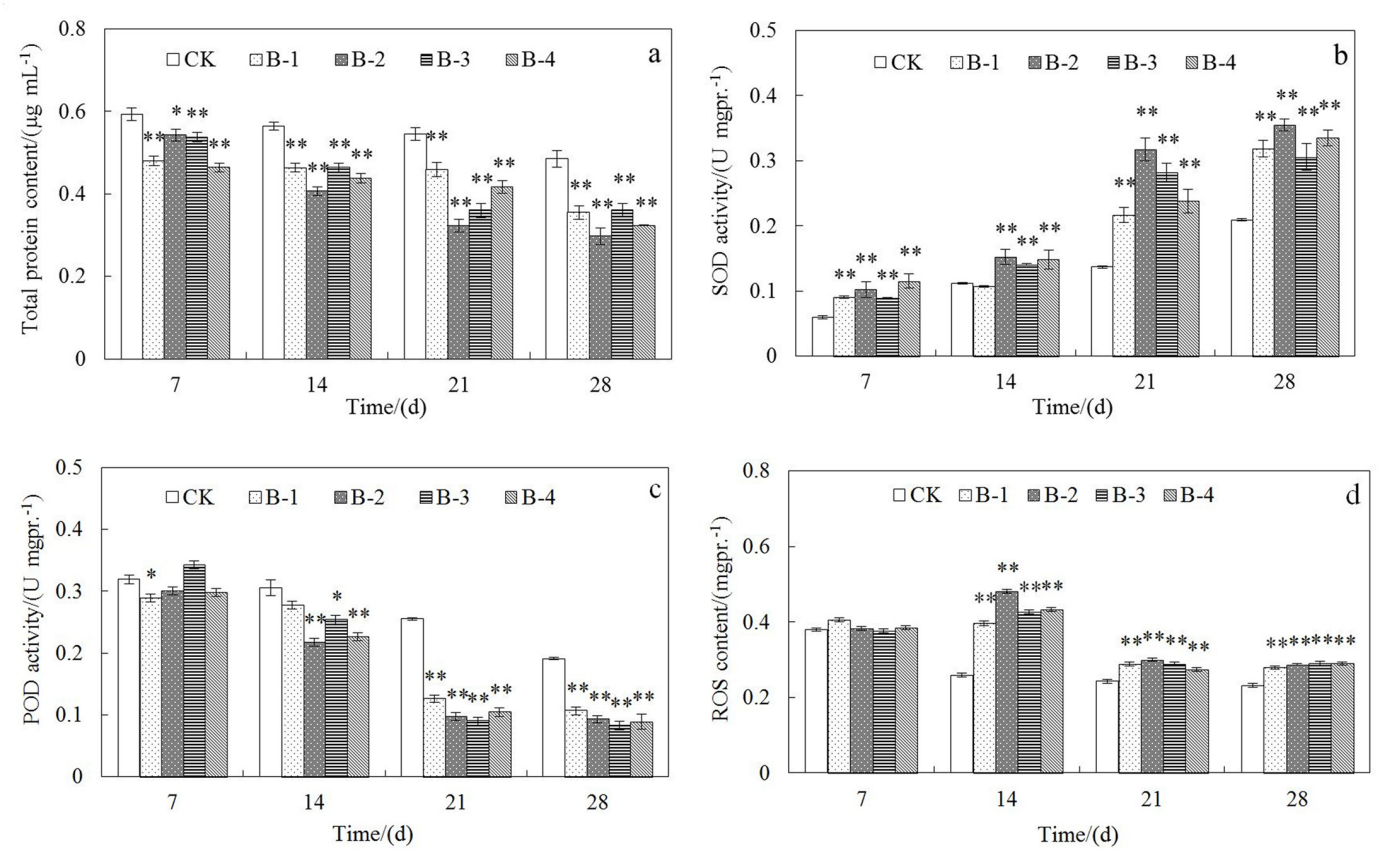
Toxicity effects of DnBP on biochemistry parameters of *E*. *fetida*. (a) Total protein content; (b) SOD activity; (c) POD activity; and (d) ROS activity were determined after treated for 7d, 14d, 21d and 28d in spiked natural soil CK, B-1, B-2, B-3, and B-4 (n = 4; error bars, SEM/mean values of standard errors). The spiked concentrations of DnBP were 0, 1, 2.5, 5, and 10 mg kg^-1^ soil. Asterisk shows significant difference at *p*<0.05 level compared to the control; double asterisks show significant difference at *p*<0.01 level compared to the control.

The total protein content in earthworms was found to be significantly higher (*p*<0.05) in control than those exposed to different concentrations of DnBP on day 7, 14, 21 and 28 (Fig 1a). The total protein contents in control treatment were all higher than 0.48 μg mL^-1^ of fresh weight (about 4.32 μg g^-1^ of the earthworm tissue), however they were all significantly lower (*p*<0.01) for earthworms that exposed for over 14 days in spiked DnBP treatments. Both the consumption of different enzymes in cellular protection under contamination stress and the inhibition of protein synthesis when earthworms were exposed to DnBP at different concentrations in soils could be the explanation of the total protein content decrease in earthworms. Both the decreasing of protein content and enzyme activities have been reported as the biomarkers developed in earthworm species [26]. As the increasing of DnBP concentrations in soil, the decrease of total protein content in earthworms was more remarkable and a slight time-dependency was observed.

#### SOD activity

In Fig 1b, the activities of SOD in earthworm *E*. *fetida* under different concentrations of DnBP, which are highly significant different (*p*<0.01) compared with the control treatment at different exposure time (except on the 14th day under 1 mg kg^-1^ DnBP), reveal the intense triggering of the antioxidant defense system. The values of SOD activity after 21 d have almost exceeded half of the control value as the increasing of time and exposure concentrations, especially in treatment of 2.5 mg DnBP kg^-1^ soil (Fig 1b). SOD catalyzes free radical superoxides conversion to peroxides and divalent oxygen and is vital in resisting against the toxicity of activated oxygen species. Augment of SOD activity may therefore protect cells from oxidative stress generated by pollutants [27]. When SOD and CAT couldn’t scavenge the generated superfluous ROS, CAT activity could be easily inhibited, so that SOD activity has been highly manifold in case of the disadvantage to CAT activity and the adjustment of different anti-oxidases [28].

#### POD activity

POD activity was inhibited at almost all concentrations of DnBP to about half the original especially after 21 days (except for the control) (Fig 1c), thus showing a similar tendency to 1,3,4,6,7,8-hexahydro-4,6,6,7,8,8-hexamethyl-cyclopenta-γ-2-benzopyran (HHCB) in a previous study of decreasing POD activity [29]. Co-substrates of H_2_O_2_ like guaiacol or ascorbate can provide electron in the decomposing of H_2_O_2_ by POD, and the mechanism of anti-oxidative defense is active due to the elevation of SOD activities. The sensitivity of antioxidant enzymes (SOD, CAT and POD) varies greatly to various oxidative stresses, indicating that their responses to oxidative attack are diver from each other.

#### ROS content

ROS contents in *E*. *fetida* were not distinctively promoted as the increasing of DnBP concentrations in spiked soils until incubation for 14 days (*p*<0.01) (Fig 1d). Organisms have developed almost perfect mechanisms to protect themselves against the toxicity of environmental pollutants by increasing ROS production and activating the antioxidant system, indicating the agreement of the results in this experiment with other studies [30]. ROS is also considered as an indicator of oxidative stress in the cellular system causing DNA damage and finally leading to the damage of different cellular organelles [31]. The protective enzymatic mechanisms including SOD, POD, and CAT and non-enzymatic mechanisms in living organisms are integrant to ROS scavenging and alleviate their deleterious effects. As the increasing of incubation days in the present study, the elimination of ROS levels might therefore be attributed to the strong depletion of SOD and POD which has utilized the accumulated ROS.

#### Flow Cytometry Assays

**Fig 2:**
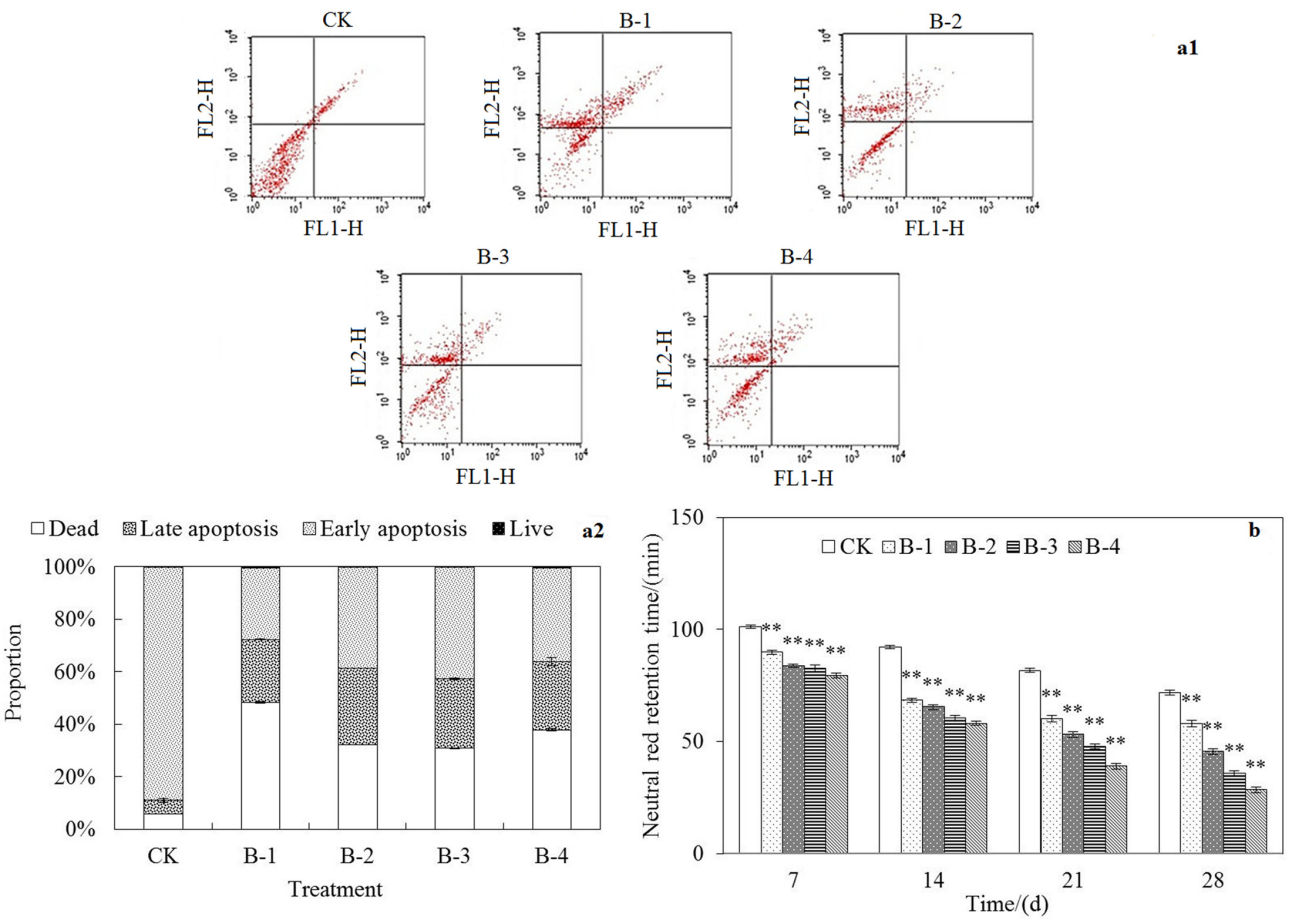
Cytotoxicity of DnBP determined by flow cytometry assays. (a) coelomocyte apoptosis (a1,a2) stained with Annexin V-FITC and PI (Upper left quadrant, necrotic cells; upper right quadrant, late/secondary apoptotic cells; bottom left quadrant, live cells; and bottom right quadrant, early/primary apoptotic cells) in the 28th day and (b) lysosomal membrane stability characterized by NRR time in extruded coelomocyte of treated *E*. *fetida* in the 7th, 14th, 21st and 28th day after exposure in spiked natural soil CK, B-1, B-2, B-3, and B-4 for 28 days (n = 4; error bars, SEM). Refer other annotates to Fig 1.

The apoptosis of coelomocytes indicates the loss of cell viability and the beginning of death. During the incubation of 28 days, the induced apoptotic coelomocyte of *E*. *fetida* was apparently replaced by the death ones compared with the control treatment but not linearly dependent with spiked concentrations of DnBP (Fig 2a1 and 2a2). AV-FITC/PI double staining method can ensure the accuracy of molecular labeling by differentiating four types of cells, namely live, early apoptotic, late apoptotic, and necrotic cells or cell debris. The death rate of coelomocyte in the control treatment was less than 6% but much higher than 40% in treatments with spiked DnBP, suggesting the severe toxicity effect and great damage of the target pollutant to earthworms.

At the initial stage of apoptosis, phosphatidylserine (PS) in cell membrane was turned from the inside to the outer surface of the cell membrane. Then the function of mitochondria began to change as the altering of membrane potential, followed by the release of some mitochondrial proteins, such as cytochrome C and Smac protein, which resulted in the activation of caspase and fragmentation of DNA. Initial apoptosis often present together with an increase in ROS, leading to enzyme damage and important intracellular organelles damage and so on, such as the effects of silver nanoparticles on earthworms [32]. The decrease of apoptosis coelomocyte could also reflect the weakening of self-repair function in cellular trauma, because it has been demonstrated that apoptosis is likely to be a normal method for biological disposal of older differentiated cells and that when worms are exposed to adverse environments, especially toxic media, down-regulation of cell populations is induced by apoptosis [33].

Flow cytometric measurement of neutral red (NR) retention in earthworm coelomocytes under the detection of fluorescence to discriminate between healthy cells and apoptotic cells has become a novel assay for studies on contamination stress [15]. Neutral red is a vital stain accumulating in lysosomes of the cells. It has been reported that the membranes of lysosomes can lose their penetrability and stability when earthworms are exposed to contaminated environments, so that NR can enter the cytoplasm and accumulate in lysosomes. The retention time of NR has been considered to be a biomarker in the assessment of toxic effects of pollutants on earthworms [34]. In all treatments under different DnBP concentrations, significantly shorten of NR retention time were observed (*p*<0.01) (Fig 2b), which indicated the obvious injury of earthworm coelomocytes after treated with soil spiked with DnBP.

### Comet Assay in Toxicity Test

**Fig 3:**
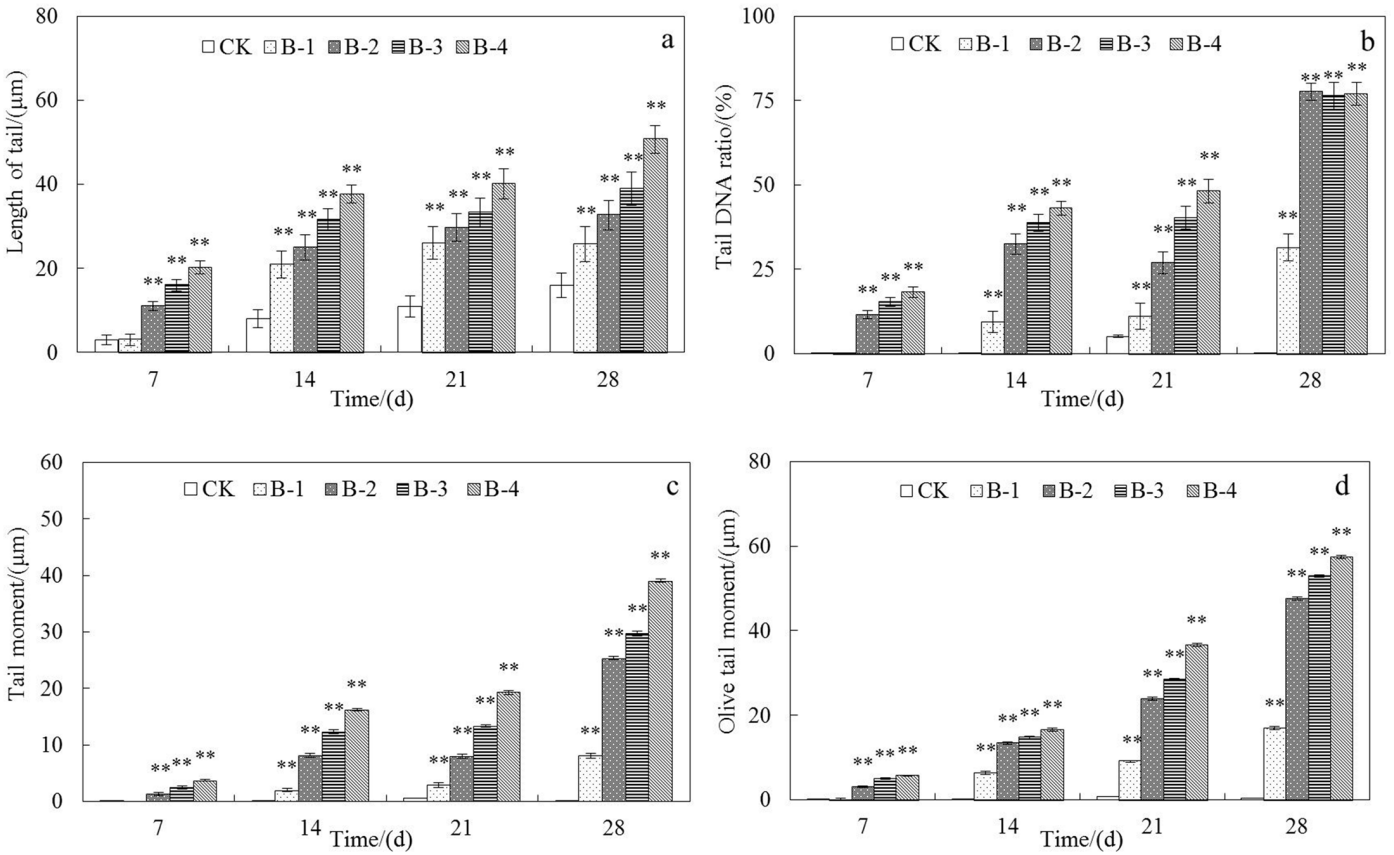
Genotoxicity of DnBP analyzed by comet assay. (a) length of tail; (b) tail DNA ratio; (c) tail moment and (d) olive tail moment of coelomocyte in treated *E*. *fetida* after treated for 7d, 14d, 21d and 28d in spiked natural soil CK, B-1, B-2, B-3, and B-4 (n = 4; error bars, SEM). Length of tail (TL) means tail length in arbitrary units; tail DNA ratio means relative ratio of DNA in the comet tail; tail moment (TM) means integrated value of DNA density multiplied by the migration distance; and Olive tail moment (OTM) means the product of the distance between the center of gravity of the head and the center of gravity of the tail and percent tail DNA. Refer other annotates to Fig 1.

According to the results in Fig 3, all these parameters suggest that when DnBP was added to soil at different concentrations the nuclei were severely damaged at the end of the incubation time. Although the length of tails was not significantly affected until the 14th day at concentrations >5 mg kg^-1^, the tail DNA % was >15% in treatments other than the control on the 7th day and finally reached about 20% by the end of the test. Clear correlations were observed between DNA damage and both the DnBP concentration of the spiked soil and the exposure time. Increasing damage to DNA during exposure was in accordance with previous results on SOD, POD and the cytotoxicity test, indicating that severe damage would occur below 5 mg kg^-1^ (*p*<0.05). Apparently, the evaluation of toxicity with DNA damage might be more sensitive than other parameters in this experiment. Some studies have indicated that DNA damage enhancement was resulted from oxidative stress, suggesting the accumulation of ROS in tissues is the main reason for the subsequent DNA damage in earthworm coelomocytes [24]. ROS has been reported to be the major source of DNA damage and it could cause strand breaks, removal of nucleotides, various modifications of the nucleotide bases and so on [35]. However, in earthworms many other anti-stress functions have developed from the long-term evolution of the species. One important biological function of metallothioneins in earthworm is to protect cells from oxidative injury and to protect DNA from damage [36]. Further studies to confirm the formation other bio-indicators such as metallothioneins and DNA adducts and cross-links could explain more about the mechanisms of this injury.

## Conclusions

In the present study almost all the indices examined were affected under a range of DnBP concentrations in spiked soils based on the special concentration from acute toxicity test and the results point to the occurrence of primary damage under DnBP stress. Strong effect of employed parameters in natural soil test emphasized the necessity of using practical environmental samples in the experiment of toxicity evaluation, just like other research in polluted water. More reliable test to stable substances in living cells should be resorted. Apoptosis of amoebocytes increased with increasing pollutant concentration after exposure for 14 days. Comet assay results were also consistent with the general regulation, indicating that substantial DNA damage was caused by exposure to DnBP in the soil. Visible damage to amoebocytes and DNA are considered more suitable and sensitive in further toxicity assessment text. More visible toxicity at the medium concentrations like 5 mg kg^-1^ soil for pollutants might indicate higher environmental risk of DnBP because they are more approaching the practical reported concentrations of target pollutants in agricultural soil. So, a soil concentration of DnBP of 5 mg kg^-1^ can be recommended for the toxicity response of *E*. *fetida* and the practical concentration of the target pollutant when comparing with other documents. The recommended concentration threshold could also serve as a reference concentration for the development of soil contamination criteria for PAE compounds. Other biomarkers should be employed to fully explain the toxicity mechanism of DnBP in soils and to seek high sensitive evaluation methods.
